# Edge-AI Enabled Wearable Device for Non-Invasive Type 1 Diabetes Detection Using ECG Signals

**DOI:** 10.3390/bioengineering12010004

**Published:** 2024-12-24

**Authors:** Maria Gragnaniello, Vincenzo Romano Marrazzo, Alessandro Borghese, Luca Maresca, Giovanni Breglio, Michele Riccio

**Affiliations:** Department of Electrical Engineering and Information Technology (DIETI), University of Naples Federico II, 80125 Naples, Italy; maria.gragnaniello@unina.it (M.G.); vincenzoromano.marrazzo@unina.it (V.R.M.); alessandro.borghese@unina.it (A.B.); luca.maresca@unina.it (L.M.); breglio@unina.it (G.B.)

**Keywords:** real-time diabetes detection, Edge-AI, deep learning, ECG, 32-bit microcontroller, spectrogram

## Abstract

Diabetes is a chronic condition, and traditional monitoring methods are invasive, significantly reducing the quality of life of the patients. This study proposes the design of an innovative system based on a microcontroller that performs real-time ECG acquisition and evaluates the presence of diabetes using an Edge-AI solution. A spectrogram-based preprocessing method is combined with a 1-Dimensional Convolutional Neural Network (1D-CNN) to analyze the ECG signals directly on the device. By applying quantization as an optimization technique, the model effectively balances memory usage and accuracy, achieving an accuracy of 89.52% with an average precision and recall of 0.91 and 0.90, respectively. These results were obtained with a minimal memory footprint of 347 kB flash and 23 kB RAM, showcasing the system’s suitability for wearable embedded devices. Furthermore, a custom PCB was developed to validate the system in a real-world scenario. The hardware integrates high-performance electronics with low power consumption, demonstrating the feasibility of deploying Edge-AI for non-invasive, real-time diabetes detection in resource-constrained environments. This design represents a significant step forward in improving the accessibility and practicality of diabetes monitoring.

## 1. Introduction

Diabetes is a chronic condition characterized by elevated blood glucose levels, known as hyperglycemia. This state is caused either by a deficiency of insulin, which is a hormone produced by the pancreas and its inefficiency in regulating glucose within the body. Symptoms of diabetes can include excessive thirst, increased urine production, fatigue, and weight loss. However, in some cases, diabetes can be asymptomatic for extended periods. Long-term complications of diabetes can affect organs and arteries, increasing the risk of cardiovascular diseases, neuropathy, retinopathy, and other health problems [1]. The most common types of diabetes are type 1, type 2, and gestational diabetes. In individuals with type 1 diabetes, the body produces minimal or no insulin due to the immune system attacking and destroying the insulin-producing cells in the pancreas. While this form of diabetes is typically diagnosed in children and young adults, it can develop at any age. Timely diagnosis is crucial for preventing or slowing the progression of complications and improving the quality of life for individuals living with this chronic disease [2]. Currently, the predominant monitoring techniques are invasive, requiring blood samples, which can cause discomfort for patients. The development of innovative, low-cost, non-invasive, and portable technologies capable of detecting diabetes at an early stage could have a significant impact on large-scale prevention efforts [3,4].

In recent years, researchers have shifted their focus towards alternative physiological markers related to diabetes, moving beyond traditional glucose measurements, which are invasive. Some are investigating signals such as photoplethysmography (PPG), while others are focusing on electrocardiograms (ECG) and the changes these signals undergo because of diabetes [5]. Although PPG-based methods have shown promise, they rely on optical techniques using LEDs and photodiodes, making them more susceptible to motion artifacts and environmental interferences in wearable devices [6]. In contrast, ECG signals do not suffer from the same issues associated with optical measurement, providing a more stable and reliable option for wearable device applications. More recently, studies have explored the use of Artificial Intelligence (AI) in the early detection of diabetes through PPG and ECG signals [5,6,7]. Both Machine Learning (ML) and Deep Learning (DL) techniques are being actively researched, providing valuable tools for identifying signal variations and gaining deeper insights into patterns and trends associated with the disease.

In this context, the paper proposes a new approach based on Edge-AI technology, where AI processes data at the edge, close to the sensors that acquire the ECG signals. Edge-AI represents a shift from traditional cloud-based processing, and has already demonstrated its potential in the biomedical field [8,9,10]. This approach reduces latency, and enhances patient privacy and real-time processing capabilities. However, it also presents challenges, such as limited computational and energy resources.

Compared with the state-of-the-art, this research distinguishes itself through the following: (i) the use of Edge-AI, which enables offline data processing unlike IoT methods; (ii) the evaluation of a benchmark on the available hardware using the STMicroelectronics platform; and (iii) the design of a custom PCB that integrates both input and output peripherals. This allows the single microcontroller to acquire ECG signals, process them using Edge-AI algorithms, and display the results remotely via Bluetooth low energy (BLE). The design of the PCB aims to demonstrate the potential to incorporate Edge-AI algorithms within existing wearable devices, enabling a non-invasive and smart early detection of diabetes. This was made possible by means of ECG signals for real-time diabetes detection through an edge computing approach.

The remaining sections of this paper are organized as follows: Section 2 provides a review of the current literature in the field. Section 3 presents the architecture of the PCB and the proposed methods. Section 4 discusses the results and a benchmark on the online available hardware. Finally, Section 5 summarizes the study and highlights the main conclusions.

## 2. Related Works

Recent research has increasingly focused on identifying diabetes through cardiovascular parameters, recognizing the high mortality rate caused by cardiovascular diseases in diabetic patients. ECGs have emerged as valuable tools due to their affordability and non-invasive nature, providing detailed insights into heart function. Key changes in the ECG associated with T1D include a flatter and more asymmetric T-wave morphology, prolonged QTc, increased QT dispersion, and variations in heart rate variability. Additionally, ST-T changes and decreased amplitudes of depolarization waves may also be observed [11,12,13].

Different approaches (summarized in Table 1) have been explored to detect diabetes through ECG signals, revealing both opportunities and limitations in this field, including considerations for the feasibility of real-time application. Andellini et al. [14] conducted a study using continuous ECG and glucose monitoring in pediatric patients with Type 1 Diabetes (T1D) [15]. By collecting data over three days using wearable devices, they observed significant correlations between glycemic states and ECG morphological features. This real-world data collection method offered valuable insights into the influence of glycemic fluctuations on ECG morphology.

Another study [16] introduced a method for classifying diabetic and non-diabetic ECG signals using 5 s single-lead ECG segments. After extracting and ranking ten features, they found that a medium tree classifier could achieve an accuracy of 87.19%. This demonstrates the feasibility of classifying diabetes based on short ECG recordings. However, the computational complexity of more advanced signal processing methods, such as the one introduced by Jain et al. [17], remains a challenge for wearable applications.

Gupta et al. explored the use of Intrinsic Time-Scale Decomposition (ITD) to extract nonlinear ECG features, achieving an accuracy of 86.90% with decision tree models [18].

In contrast, in Ref. [19], DL techniques are applied, developing a 10-layer model to detect hyperglycemia. They present a different feature extraction approach based on slope, temporal, and amplitude characteristics. Their method reduced the feature set by 97% compared to using a full ECG heartbeat, achieving an area under the ROC curve (AUC) of 94.53%. Although their results were promising, the method is more suitable for clinical set-up. Further studies like [20] spectrogram-driven 2D-CNNs are used to analyze ECG data, achieved high accuracy (96.90%), but they face challenges related to the limited number of patients. Meanwhile, Yildirim et al. explored heart rate signals using transfer learning with models like DenseNet, AlexNet and ResNet, achieving high accuracy and sensitivity (97.20% and 100%, respectively) [21].

In conclusion, while ECG-based diabetes detection has made notable strides, several challenges persist. Traditional methods such as feature extraction are advantageous for their simplicity, while DL models deliver superior accuracy and generalization capabilities. However, critical issues such as limited dataset sizes, feature selection, and real-time applicability need to be addressed for these systems to find widespread use not only in clinical settings, but also for remote continuous monitoring. Such applications could further reduce invasiveness in patients’ daily lives, providing a seamless integration into health management routines. A significant hurdle is the accessibility of large and diverse datasets, as many are privately held and not available for wider research, limiting the scope of model training. Moving forward, efforts should focus on creating more generalized models that can handle real-time monitoring and diagnosis across broader populations and clinical conditions. This shift would make it easier to implement these systems for everyday use, enhancing both the early detection of diabetes and ongoing management of the disease in a more user-friendly, non-intrusive manner.

## 3. Materials and Methods

In this section, a detailed description of the selected hardware components and PCB design is provided, followed by an explanation of the proposed method, which includes the use of spectrogram analysis and the design of a 1D-CNN.

### 3.1. Hardware Components Selection

**Figure 1:**
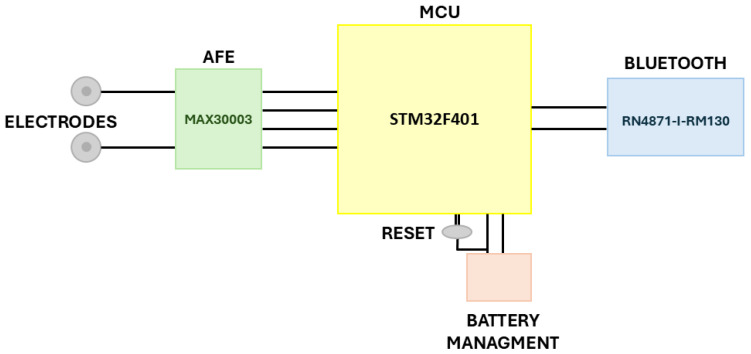
Schematic block diagram of the PCB design. The main components include the section for the Analog Front-End (AFE) using the MAX30003, the Microcontroller Unit (MCU) based on the STM32F401, and the output section utilizing Bluetooth low energy (BLE) for communication.

The hardware design process began with the selection of key components essential for the acquisition and processing of ECG signals. The main goal was a miniaturized and lightweight device, suitable for continuous monitoring, while maintaining high accuracy in ECG signal acquisition thanks to the use of a medical grade device. Figure 1 shows a high-level block diagram of a PCB design.

The microcontroller unit (MCU) chosen for this design was the STM32F401 (STMicroelectronics, Geneva, Switzerland) [22], a 32-bit ARM^®^ Cortex^®^-M4 processor [23], clocked at 84 MHz. It features 512 kB of flash memory and 96 kB of RAM. Despite being part of the high-performance STM32F4 family [24], this MCU was found to be well-suited for continuous monitoring applications, balancing both processing power and energy consumption; further details are described in Section 4. The system is powered by a battery, for which an adapter was specifically designed to ensure efficient power management. The device is equipped with specialized components for ECG signal acquisition and processing, featuring the MAX30003 (Analog Devices, Norwood, MA, USA) for precise signal acquisition and the RN4871-I-RM130 (Microchip Technology, Chandler, AZ, USA) module for output communication.

The MAX30003 is a medical-grade integrated chip produced by Analog Devices, optimized for real-time single-lead ECG monitoring [25]. It includes features such as ESD protection, EMI filtering, internal lead biasing, and ultra-low power lead-off detection during standby. The chip offers high impedance input, low noise, configurable gain, and several filtering options, along with a high-resolution analog-to-digital converter (ADC). These characteristics enable the MAX30003 to capture high-quality ECG signals with minimal interference and power consumption, outperforming devices like the AD8232 (Analog Devices, Norwood, MA, USA) [26]. Unlike the AD8232, which requires external components to achieve similar functionality, the MAX30003 integrates numerous features directly into the chip, simplifying circuit design and reducing overall costs. Notably, it includes built-in algorithms for RR peak detection, significantly lowering the computational burden on the MCU. In contrast, the AD8232 is limited to sampling the ECG signal, leaving all computational tasks to the MCU. This makes the MAX30003 a more versatile and efficient solution, particularly for advanced cardiac monitoring applications.

**Figure 2:**
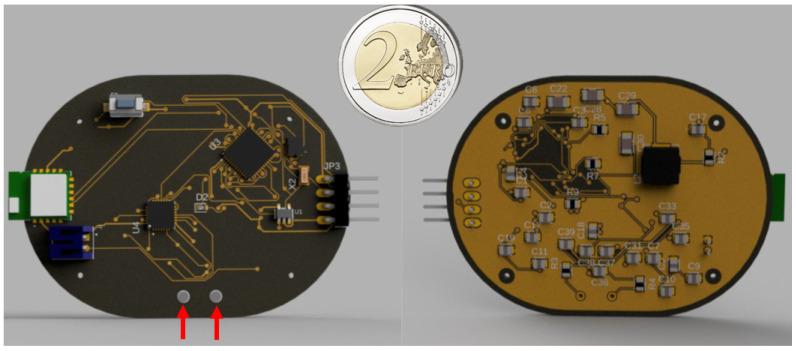
Rendering of the top and bottom layers of the custom PCB. On the left side, the electrodes are highlighted.

For Bluetooth communication, the RN4871-I-RM130 module was selected, utilizing Bluetooth low energy (BLE) technology to enable short-range wireless communication with low power consumption [27]. This module supports the Bluetooth 4.2 standard, providing improved data transfer rates, extended range, and better energy efficiency. It communicates with the MCU via the UART interface, making it a popular choice for wearable devices where low-power wireless connectivity is essential, as in the current design. The final design (Figure 2) has dimensions of 60 mm × 45 mm.

### 3.2. Proposed Method

**Figure 3:**
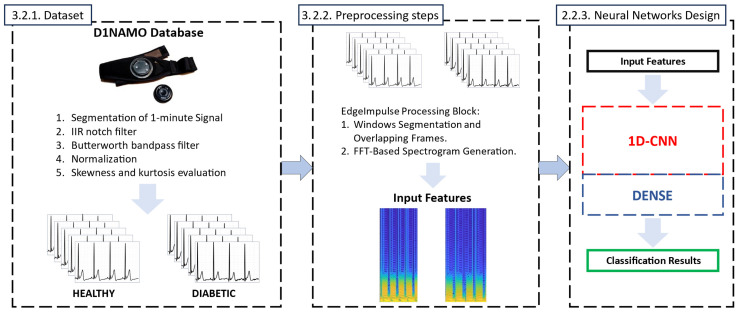
The overall procedure began with the creation of a useful dataset by D1NAMO. These data were then processed through spectrogram analysis, followed by CNN inference, which was used to display the results.

The overall procedure is schematized in Figure 3.

#### 3.2.1. Dataset

The D1NAMO database was employed in this study, representing a multimodal dataset aimed at researching the non-invasive management of type 1 diabetes (T1D) [28,29]. The sample comprised 20 healthy subjects and 9 patients diagnosed with T1D. This dataset includes various physiological measurements such as ECG signals, respiration data, accelerometer readings, glucose levels, and annotated food images. Data collection was conducted using the Zephyr BioHarness 3 (Zephyr Technology, Annapolis, MD, USA) handheld device [30] under real-world conditions, leading to the presence of significant noise and missing data.

For our analysis, only the visibly clear 1 min segments of the ECG signals were used following a comprehensive review of the dataset. Initially, the signals were segmented into 1 min intervals. Subsequently, preprocessing steps were applied to eliminate unwanted artifacts; the signals were denoised using an IIR notch filter to remove 50 Hz interference, followed by a fourth-order Butterworth bandpass filter with a frequency range of 0.5–40 Hz. Finally, the cleaned segments were normalized, and statistical measures such as skewness and kurtosis were evaluated. These metrics allowed us to establish thresholds for distinguishing clean signals from those classified as noisy, thereby enhancing the reliability of the dataset for the subsequent analyses.

This approach was informed by the analysis of ECG signals from other studies, such as those in [8,9]. By studying the statistical parameters of signals rated clean in those studies, we established acceptable ranges for skewness and kurtosis. In our case, the acceptable range for skewness was [0 5], and for kurtosis, it was [3 30]. The algorithm processes 1 min ECG signals by calculating skewness and kurtosis on non-overlapping 1 s segments. A segment is classified as “clean” if its values fall outside the acceptable range no more than three times. Table A1 in Appendix A provides a concise pseudocode representation of the proposed threshold-based dataset cleaning algorithm.

A critical aspect of building the dataset—and consequently the entire study—was the limited number of diabetic patients, which posed a high risk of overfitting. Specifically, there was a concern that the NN might learn to identify individual patients from their ECG signals, rather than detecting patterns associated with T1D. To mitigate this risk, the dataset was rigorously partitioned to ensure a patient-independent split. No patient included in the training phase was present in the validation or testing phases. The dataset was divided based on the number of acceptable ECG frames per patient to achieve a partition close to the standard 80/20 split. Given the larger number of healthy patients compared to diabetic ones, the dataset naturally included more ECG frames from healthy individuals. To address this imbalance, a randomized selection of frames per healthy patient was performed to create a dataset that was as balanced as possible. The training set was sufficiently large to capture the variability in ECG signals, while the testing set provided an unbiased evaluation using a distinct group of patients. This careful partitioning and balancing approach ensured that the model focused on detecting T1D-related patterns rather than overfitting to individual patient characteristics or being biased toward the majority class. The exact distribution of samples for each phase and the number of segments per patient are provided in Table A2 and Table A3 of Appendix A.

**Figure 4:**
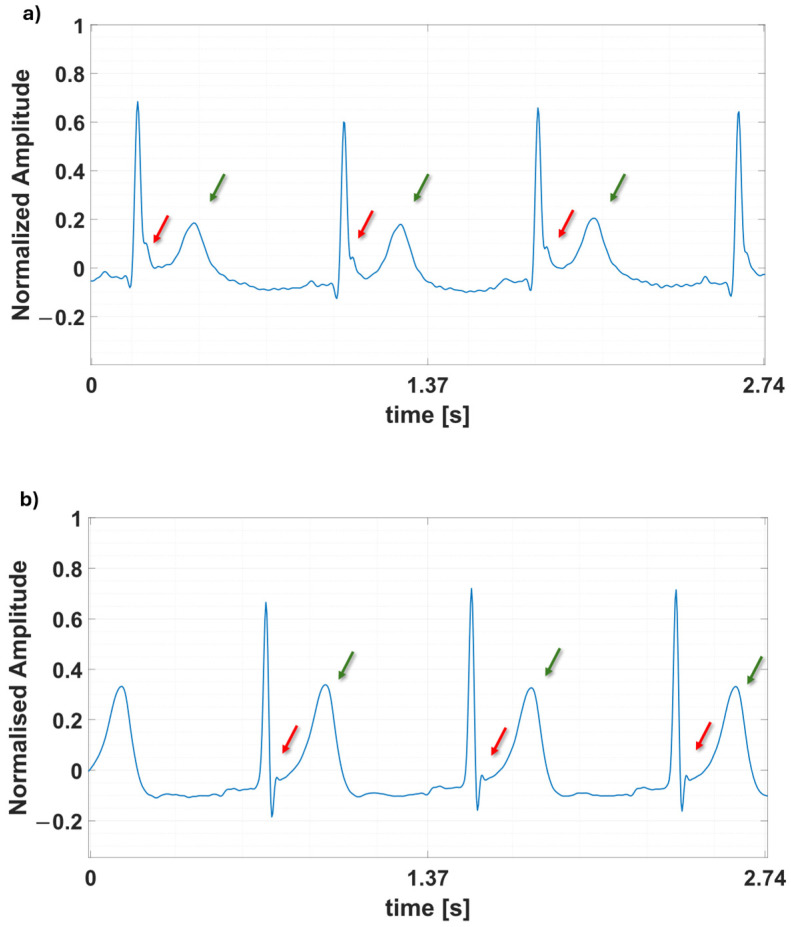
Example waveforms of (**a**) a diabetic ECG signal and (**b**) a healthy ECG.

This approach enabled the creation of a final dataset encompassing 83 h and 15 min of acquisition, comprising 2497 diabetic samples and 2498 healthy samples. Figure 4 presents an example of ECG readings for each label in the dataset. The figure highlights key differences, such as a deviation in the ST segment and an asymmetric T-wave morphology in the diabetic ECG, which contrasts with the typical T-wave shape observed in healthy individuals.

#### 3.2.2. Preprocessing Steps

While the dataset was constructed using MATLAB R2023a, the entire preprocessing and NN design and training process was carried out using the Edge Impulse development platform [31]. The designed Machine Learning pipeline consists of a processing block and a learning block, applied on 5 s segments with a 1 s overlap. The processing block computes a spectrogram for each segment, and the learning block is used for classification. The spectrogram configuration included a frame length of 0.7 s, a stride of 10% (0.07 s), and an FFT length of 256, with a noise floor set to −50 dB. This configuration results in a total of 60 frames per segment, yielding 7740 features in total.

The spectrogram provides a visual representation of signal frequency intensities over time. By dividing a signal into overlapping time windows and applying the Fourier Transform to each segment, it delivers localized frequency information, capturing dynamic changes in signal components. The clarity and simplicity of interpretation make the spectrogram one of the most valuable tools for analyzing time frequencies. For resource-constrained systems like microcontrollers, the spectrogram offers significant advantages. It is computationally less demanding compared to other frequency-domain techniques, such as the Discrete Wavelet Transform (DWT), which remains widely used in ECG signal analysis [32]. DWT maps a signal onto a time-scale plane using scaled and shifted versions of a principal wavelet [33]. However, its multiscale approach requires extensive computational resources due to its complex operations. Moreover, wavelet analysis is highly sensitive to the choice of the principal wavelet, which can heavily influence results and limit its adaptability in certain applications.

#### 3.2.3. Neural Networks Design

**Figure 5:**
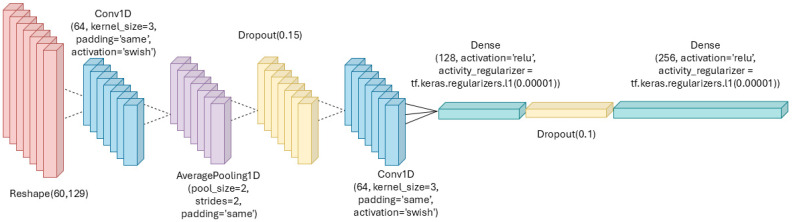
Schematic representation of the neural network design. The structure consists of input layers for ECG data, followed by key processing layers, leading to the final classification output.

The designed NN is a convolutional model consisting of two 1D convolutional layers (1D-CNN) and two dense layers, as illustrated in Figure 5. Although the input to the network is an image (the spectrogram of the signal) rather than a one-dimensional signal, 1D-CNN layers were employed instead of 2D-CNN. This decision was primarily driven by hardware constraints, as the model needs to have low memory and energy consumption, given that it will be deployed on a single microcontroller capable of acquiring, preprocessing, and transmitting results via Bluetooth. Furthermore, the use of 1D-CNN layers is appropriate given the data structure; each spectrogram frame is 0.7 s long, which is sufficient to capture a single heartbeat. The 90% overlap between frames allows the model to account for variations between consecutive beats, preserving the consistency and coherence of the medical information within each frame. By analyzing the frames separately using 1D-CNN layers, it is possible to extract relevant features without losing critical information for classification. The model was trained for 500 epochs with a learning rate of 5 × 10^4^ and a batch size of 128. Early stopping was implemented to monitor the loss values and prevent overfitting. The Adam optimizer was used for model training. Further details on the architecture are provided in Figure 5.

The Swish activation function was used for the 1D-CNN layers instead of the classical ReLU thanks due to its advantages in handling complex data more efficiently, and avoiding issues such as the “dying ReLU” problem. Swish is characterized by a smooth, sigmoid-like curve that allows for a more gradual and continuous increase in output, which helps prevent neurons from becoming inactive during training—a common issue with ReLU, where neurons can output zero and fail to contribute to learning [34]. Additionally, average pooling was employed instead of max pooling, as it preserves more contextual information by considering all values within a region. This reduces the risk of losing critical details, making the network more robust to small variations in the input and providing a more stable representation of the feature map. Lastly, dropout was incorporated to mitigate potential overfitting, especially given the limited number of diabetic patients in the dataset.

## 4. Results

**Figure 6:**
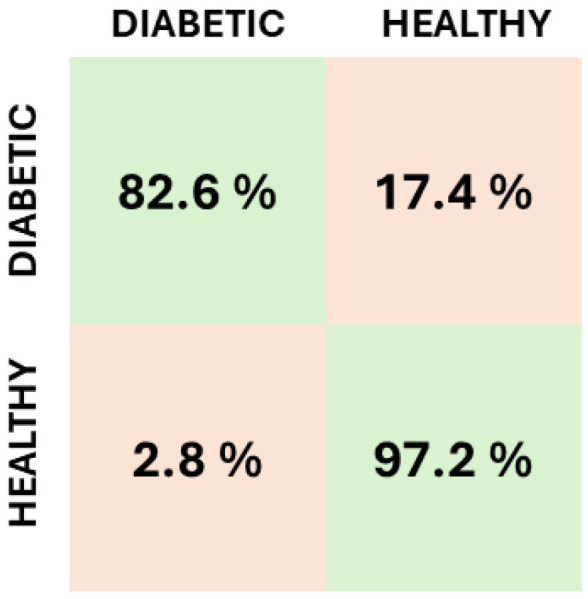
Confusion matrix.

The NN, optimized using the EON compiler [35] instead of the typical TensorFlow Lite approach, achieved an accuracy of 89.52% with an average precision of 0.91, an average recall of 0.90, and an AUC of 0.90. Figure 6 illustrates the confusion matrix obtained on the test dataset, while Table 2 provides a summary of the Edge Impulse report on model validation.

According to the Edge Impulse training report, the quantized model for a Cortex-M4F architecture at 80 MHz requires 352.2 kB of flash memory and 33.2 kB of RAM, with a latency of 103 ms for spectrogram generation and 77 ms for classifier inference. In contrast, the unoptimized model requires 1.2 MB of flash memory, making it unsuitable for deployment on high-resource-constrained microcontrollers. The application of optimization techniques, such as quantization, allowed for a significant reduction in model size and memory usage, while maintaining comparable accuracy. In this case, using the EON compiler resulted in the same accuracy, with 20% less RAM and 70% flash consumption. Quantization or pruning are essential techniques in the fields of Edge AI and TinyML, focusing on optimizing NNs for memory efficiency and power consumption, often with a minimal impact on performance metrics like accuracy.

This work, compared to the studies summarized in Table 1, which intentionally rely on different methods for processing ECG signals, may not achieve the same high accuracy as those reported in [17,19,20,21]. However, it provides a comprehensive initial study on the feasibility of detecting diabetes near the site of ECG signal acquisition. The results demonstrate both the effectiveness and practicality of this approach. The limitations of the dataset are well known, particularly the small cohort of patients. Future efforts aim to validate the model on a larger dataset to further enhance performance. Throughout this process, our focus remains on ensuring that the system functions effectively on low-resource wearable devices, with minimal energy consumption to maximize battery life and support prolonged usage.

In this regard, the selected microcontroller belongs to the F4 series, and it was chosen from several 32-bit microcontrollers across different ARM architectures, including M4, M7, and M33. Specifically, the F7, H7, H5, U5, G4, and F4 were benchmarked using the ST Edge AI Developer Cloud (2.0.0) [36] tool. These microcontrollers were ranked based on a figure of merit (FOM), as described by Equation (1), which summarizes the trade-off between power dissipation and inference time (*t_i_*). While a microcontroller with a higher clock frequency is expected to produce results with lower latency (*t_i_ ∝ f_clk_*), it is also expected to have higher energy consumption (power dissipation *∝ f_clk_^2^*).

(1) $$FOM=\frac{1}{t_{i}\;\cdot \;{f_{clk}}^{2}}$$

**Figure 7:**
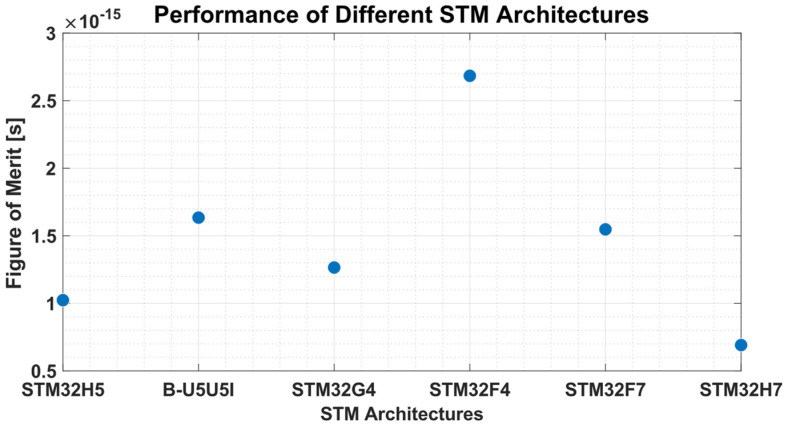
Graphical results from the ST Edge AI Developer Cloud following the benchmark test.

As evident from Figure 7, despite the F4 series having the slowest clock frequency and consequently the slowest inference time, as reported in Table 3, still achieved the highest FOM by a significant margin. It is noteworthy that the F4 series can complete the task in a very short time (52.80 ms), making it suitable for everyday use, as this interval is practically imperceptible. Considering its compact size and the dedicated electronics, the F4 series remains the optimal choice, striking a balance between performance and power consumption.

It is noteworthy that, prior to running the benchmark, an additional optimization in RAM usage was performed using the platform, allowing for a reduction to 23 kB of RAM. A summary of all optimization results is provided in Table 4, showing the impact of each optimization step. In future work, the aim is to utilize the ST Edge AI Developer Cloud further for quantization and NNs optimization, specifically targeted at microcontrollers.

## 5. Conclusions

This study demonstrates the feasibility of using Edge-AI for non-invasive type 1 diabetes detection through ECG signal analysis on a wearable device. The research employs a spectrogram-based preprocessing technique combined with a 1D-CNN to extract relevant features from ECG data. The quantization technique has been applied to ensure efficient deployment on resource-constrained devices based on a single 32-bit microcontroller. This optimization significantly reduced memory usage without compromising the model’s accuracy when compared to its initial performance. The final model achieves an accuracy of 89.52%, with an average precision and recall of 0.91 and 0.90, respectively, and a memory footprint of 347 kB flash and 23 kB RAM. An extensive benchmark has been conducted on various 32-bit microcontrollers, demonstrating the STM32F4 series as a viable option for balancing performance and power consumption. The design of a custom PCB enabled the integration of input and output peripherals, allowing the microcontroller to acquire ECG signals, perform local processing based on the Edge-AI approach, and communicate results through BLE. The thresholds described in Section 3.2.1 used for dataset cleaning also provide a secondary benefit, as they can be employed to assess the quality of acquired ECG signals in real-world environments. This reduces incorrect results and minimizes energy consumption, as CNN is triggered only when acceptable signal quality is detected. Furthermore, the filtering and protection mechanisms of the MAX30003, including ESD protection, EMI filtering, and ultra-low power lead-off detection, contribute to the proper functionality of the system. These features ensure high-quality ECG signal acquisition with minimal interference, optimizing the system’s performance and reliability in real-world scenarios. Future work will focus on improving the handling of signal durations to explore the feasibility of obtaining results from a single heartbeat, and further optimizing the model to enhance accuracy and reduce power consumption.

## Figures and Tables

**Table 1:** Overview of related works focusing on ECG-based diabetes detection.

Article	Method	Signal	Results	Suitable for Wearable Device?
[16]	Medium Tree Classifier	ECG	Acc = 87.19%	It is possible, but real-time performance was not evaluated.
[17]	TQWT and SVM Classifier	ECG	Acc = 91.50%	Although TQWT is more flexible for signal analysis, it requires higher computational complexity and resources, making it unsuitable for low-resource MCUs.
[18]	ITD and Fine Decision Classifier	ECG	Acc = 86.90%	It is possible, but the ITD algorithm requires optimization to work within the computational constraints of an MCU.
[19]	Deep Learning Classifier	ECG	AUC = 94.53%	Not suitable for an MCU with limited memory due to the deep NN, and the noise-sensitive feature extraction may cause errors in real-time.
[20]	Spectrogram and 2D-CNN	ECG	Acc = 96.90%	Despite its significant results, this technique demands excessive computational and memory resources.
[21]	Spectrogram and Pre-Trained Deep Models	HR	Acc = 97.20%	Pre-trained networks require an excessive number of parameters, as they are not designed to operate with limited resources.

**Table 2:** Summary of the Edge Impulse report.

Parameter	Result
AUC	0.90
Weighted average precision	0.91
Weighted average recall	0.90
Weighted average F1 score	0.90

**Table 3:** Summary of benchmark results based on the inference time required for each microcontroller.

Method	Clock Frequency (MHz)	Inference Time (ms)
STM32H5	250	15.63
B-U5U5I	160	23.90
STM32G4	170	27.35
**STM32F4**	**84**	**52.80**
STM32F7	216	13.85
STM32H7	480	6.28

**Table 4:** Summary of optimization results, starting from 63.1 kB of RAM and 1.2 MB of flash usage.

Method	Memory Footprint Obtained
EON Compiler	RAM = 33.2 kB FLASH = 352.2 kB
ST Edge AI Developer Cloud	RAM = 23 kB FLASH = 347 kB

## Data Availability

Dataset available on request from the authors.

## Appendix A

**Table A1:** Overview of the threshold-based algorithm for dataset cleaning.

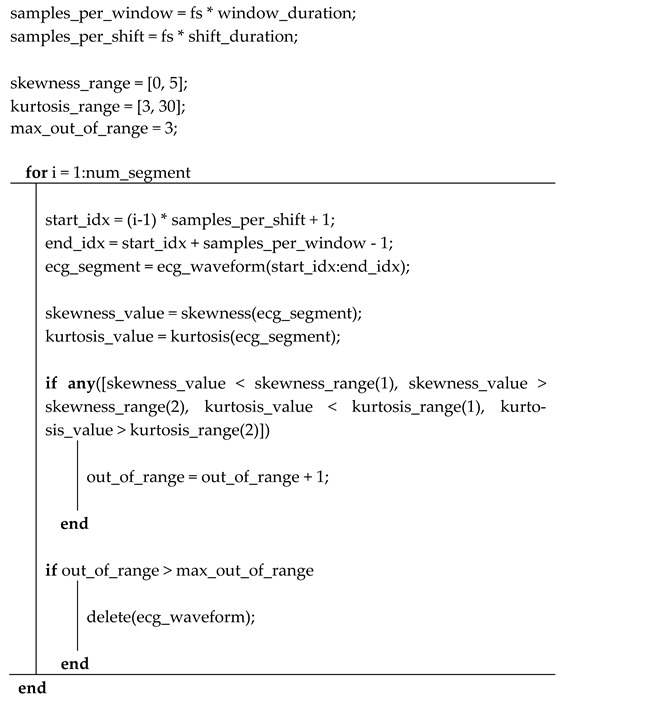

**Table A2:** ECG frames for training dataset.

Patient	Number of 1 min ECG Frames	Label
7	452	Healthy
10	28	Healthy
11	274	Healthy
12	83	Healthy
13	120	Healthy
14	118	Healthy
15	3	Healthy
16	199	Healthy
17	78	Healthy
18	18	Healthy
19	19	Healthy
20	20	Healthy
2	202	Diabetic
4	760	Diabetic
6	619	Diabetic
8	85	Diabetic

**Table A3:** ECG frames for validation dataset.

**Patient**	**Number of 1 min ECG Frames**	**Label**
1	110	Healthy
2	152	Healthy
3	116	Healthy
4	77	Healthy
5	93	Healthy
8	144	Healthy
9	93	Healthy
1	396	Diabetic
3	62	Diabetic
5	27	Diabetic
7	349	Diabetic
9	162	Diabetic

## References

[1] Diabetes. https://www.who.int/health-topics/diabetes#tab=tab_1.

[2] What Is Diabetes?—NIDDK, National Institute of Diabetes and Digestive and Kidney Diseases. https://www.niddk.nih.gov/health-information/diabetes/overview/what-is-diabetes.

[3] Laha S., Rajput A., Laha S.S., Jadhav R. (2022). A Concise and Systematic Review on Non-Invasive Glucose Monitoring for Potential Diabetes Management. Biosensors.

[4] Welch G., Balder A., Zagarins S. (2015). Telehealth Program for Type 2 Diabetes: Usability, Satisfaction, and Clinical Usefulness in an Urban Community Health Center. Telemed. E-Health.

[5] Zanelli S., Ammi M., Hallab M., El Yacoubi M.A. (2022). Diabetes Detection and Management through Photoplethysmographic and Electrocardiographic Signals Analysis: A Systematic Review. Sensors.

[6] Andreozzi E., Sabbadini R., Centracchio J., Bifulco P., Irace A., Breglio G., Riccio M. (2022). Multimodal Finger Pulse Wave Sensing: Comparison of Forcecardiography and Photoplethysmography Sensors. Sensors.

[7] Piet A., Jablonski L., Daniel Onwuchekwa J.I., Unkel S., Weber C., Grzegorzek M., Ehlers J.P., Gaus O., Neumann T. (2023). Non-Invasive Wearable Devices for Monitoring Vital Signs in Patients with Type 2 Diabetes Mellitus: A Systematic Review. Bioengineering.

[8] Gragnaniello M., Borghese A., Marrazzo V.R., Maresca L., Breglio G., Irace A., Riccio M. (2024). Real-Time Myocardial Infarction Detection Approaches with a Microcontroller-Based Edge-AI Device. Sensors.

[9] Gragnaniello M., Balbi F., Martellotta G., Borghese A., Marrazzo V.R., Maresca L., Breglio G., Irace A., Riccio M. Edge-AI on Wearable Devices: Myocardial Infarction Detection with Spectrogram and 1D-CNN. Proceedings of the 2024 IEEE 22nd Mediterranean Electrotechnical Conference, MELECON 2024.

[10] Gragnaniello M., Borghese A., Marrazzo V.R., Breglio G., Irace A., Riccio M. (2024). A Microcontroller-Based System for Human-Emotion Recognition with Edge-AI and Infrared Thermography. Lect. Notes Electr. Eng..

[11] Isaksen J.L., Graff C., Ellervik C., Jensen J.S., Andersen H.U., Rossing P., Kanters J.K., Jensen M.T. (2018). Type 1 diabetes is associated with T-wave morphology changes. The Thousand & 1 Study. J. Electrocardiol..

[12] The ECG in Diabetes Mellitus. https://www.ahajournals.org/doi/epub/10.1161/CIRCULATIONAHA.109.897496.

[13] Kittnar O. (2015). Electrocardiographic changes in diabetes mellitus. Physiol. Res..

[14] Andellini M., Castaldo R., Cisuelo O., Franzese M., Haleem M.S., Ritrovato M., Pecchia L., Schiaffini R. (2024). Are the variations in ECG morphology associated to different blood glucose levels? implications for non-invasive glucose monitoring for T1D paediatric patients. Diabetes Res. Clin. Pract..

[15] Andellini M., Haleem S., Angelini M., Ritrovato M., Schiaffini R., Iadanza E., Pecchia L. (2023). Artificial intelligence for non-invasive glycaemic-events detection via ECG in a paediatric population: Study protocol. Health Technol..

[16] Jain A., Verma A., Verma A.K. (2023). Non-invasive and Automatic Identification of Diabetes Using ECG Signals. Int. J. Electr. Electron. Res..

[17] Jain A., Verma A., Verma A.K., Bajaj V. (2024). Tunable Q-factor wavelet transform based identification of diabetic patients using ECG signals. Comput. Methods Biomech. Biomed. Engin..

[18] Gupta K., Bajaj V. (2022). A Robust Framework for Automated Screening of Diabetic Patient Using ECG Signals. IEEE Sens. J..

[19] Cordeiro R., Karimian N., Park Y. (2021). Hyperglycemia Identification Using ECG in Deep Learning Era. Sensors.

[20] Cisuelo O., Haleem M.S., Hattersley J., Pecchia L., Badnjević A., Gurbeta Pokvić L. (2024). Spectrogram-Driven Convolutional Neural Network for Real-Time Non-invasive Hyperglycaemia Detection in Paediatric Type-1 Diabetes via Wearable Sensors. MEDICON’23 and CMBEBIH’23.

[21] Yildirim O., Talo M., Ay B., Baloglu U.B., Aydin G., Acharya U.R. (2019). Automated detection of diabetic subject using pre-trained 2D-CNN models with frequency spectrum images extracted from heart rate signals. Comput. Biol. Med..

[22] NUCLEO-F401RE-STM32 Nucleo-64 Development Board with STM32F401RE MCU, Supports Arduino and ST Morpho Connectivity—STMicroelectronics. https://www.st.com/en/evaluation-tools/nucleo-f401re.html.

[23] Arm Cortex-M4—Microcontrollers—STMicroelectronics. https://www.st.com/content/st_com/en/arm-32-bit-microcontrollers/arm-cortex-m4.html.

[24] STM32F4—ARM Cortex-M4 High-Performance MCUs—STMicroelectronics. https://www.st.com/en/microcontrollers-microprocessors/stm32f4-series.html.

[25] MAX30003 Datasheet and Product Info|Analog Devices. https://www.analog.com/en/products/max30003.html#product-overview.

[26] ad8232.pdf. https://www.analog.com/media/en/technical-documentation/data-sheets/ad8232.pdf.

[27] RN4870_71_Bluetooth_Low_Energy_Module_DS50002489-3002868.pdf. https://www.mouser.it/datasheet/2/268/RN4870_71_Bluetooth_Low_Energy_Module_DS50002489-3002868.pdf.

[28] Dubosson F., Ranvier J.-E., Bromuri S., Calbimonte J.-P., Ruiz J., Schumacher M. (2018). The open D1NAMO dataset: A multi-modal dataset for research on non-invasive type 1 diabetes management. Inform. Med. Unlocked.

[29] D1NAMO ECG Glucose Data. https://www.kaggle.com/datasets/sarabhian/d1namo-ecg-glucose-data.

[30] Zephyr BioHarness—Zephyr—Catalogo PDF|Documentazione tecnica|Brochure. https://pdf.medicalexpo.it/pdf-en/zephyr/zephyr-bioharness/83995-97427.html#open684688.

[31] Edge Impulse. https://edgeimpulse.com/.

[32] Pradhan B.K., Neelappu B.C., Sivaraman J., Kim D., Pal K. (2023). A Review on the Applications of Time-Frequency Methods in ECG Analysis. J. Healthc. Eng..

[33] Zyout A., Alquran H., Mustafa W.A., Alqudah A.M. (2023). Advanced Time-Frequency Methods for ECG Waves Recognition. Diagnostics.

[34] Ramachandran P., Zoph B., Le Q.V. Searching for Activation Functions. https://openreview.net/forum?id=SkBYYyZRZ.

[35] EON Compiler|Edge Impulse Documentation. https://docs.edgeimpulse.com/docs/edge-impulse-studio/deployment/eon-compiler.

[36] ST Edge AI Developer Cloud, STMicroelectronics—STM32 AI. https://stm32ai.st.com/st-edge-ai-developer-cloud/.

